# Carers’ concerns about their older persons (Carees) at risk of falling – a mixed-methods study protocol

**DOI:** 10.1186/s12913-018-3632-6

**Published:** 2018-10-26

**Authors:** Seng Giap Marcus Ang, Anthony Paul O’Brien, Amanda Wilson

**Affiliations:** School of Nursing and Midwifery, Faculty of Health and Medicine, University of Newcastle, Callaghan, NSW 2308 Australia

**Keywords:** Carer, Older person, Fall concern, Fall risk, Fear of falling, Mixed methods

## Abstract

**Background:**

When dependent older persons (carees) experience a fall at home, their carers worry that they will fall again. This ongoing concern affects the carers’ wellbeing, perception of burden and can potentially change care arrangements. Previous research has focused on carers of high fall risk older persons with stroke, dementia or Parkinson’s disease. However, little is known about the carers’ concerns for carees at risk of falling generally; and there is no validated instrument to measure this concern. This study aims to explore carers’ fall concern about carees at risk of falling and the development of an instrument to measure this concern.

**Methods:**

This study utilises an exploratory sequential design in the development of an instrument to measure carers’ concerns. Phase One will explore carers’ fall concern using a descriptive qualitative approach. Phases Two and Three will involve expert review, pilot testing and field testing of the instrument. Twenty participants will be recruited by purposive sampling in phase one, and convenience sampling of 50 and 250 participants respectively, in Phases Two and Three. The participants will be recruited from research volunteer registers and local hospital outpatient clinics.

Participants will be 18 years old and older and the main carer of an older person. Participants will be interviewed about their concerns about falls. Inductive content analysis will be used to analyse interviews and develop items for the instrument. The psychometric properties of the raw instrument will be tested using an online survey. This study has received ethics approval from the Hunter New England Human Health Research Ethics Committee.

**Discussion:**

This study aims to provide greater depth of understanding about the psychological concerns and emotional burden related to carees’ falls for carers. Quantifying carers’ concerns will provide a context for interventions to assist and support carers and in the greater vigilance of monitoring the falling incidence of carees.

## Background

In this paper, the term “caree” is used to refer to an older person, who is dependent on someone to assist them in their daily activities. This term is used to standardise the current variation of terms and euphemisms (such as older people, older persons, elders, family members, and loved ones).

Falls are the second leading cause of unintentional injury deaths internationally [1]. About 646,000 people die from falls each year with those aged above 65 suffering the highest number of fatal falls [1]. Hospitalisations due to injuries sustained from falls are also common among carees. In Australia, 126,000 people aged 65 and above hospitalised due to injuries between 2011 and 2012 [2]. Of these, 77% sustained injuries due to a fall and the rate of injuries increased with age [2]. More than twice as many women than men were hospitalised, and majority of the falls occurred at home (49.6%), followed by residential institutions (22.5%) [2]. According to the Australian Bureau of Statistics, the proportion of people aged above 65 years increased from 11.9 to 15.0% between 1995 and 2015, and is projected to increase by another 1.1% by year 2020 [3]. With the population ageing, falls are an imminent public healthcare issue among carees, especially those living at home [4].

Falls can cause adverse psychological impact on carees, increased fear of falling again, decreased self-efficacy, and confidence in balance [5]. Up to 85% of carees living at home experience a fear of falling associated with activity restriction/avoidance [5–7], leading to decreased physical and mental performance and poorer quality of life [6, 8]. After a fall, carees with fear of falling can become more dependent, have a higher risk of falling, and are at increased risk of being admitted to long-term institutional care [6, 9]. The concepts of fear of falling in this group has been the subject of many studies looking to quantify it using different instruments [10]. However, these instruments are mostly designed to measure the psychological effect of falling among older people. The instruments are limited to questions about activity restriction, or the types of activities the respondents may perform, and do not provide an understanding about the burden that carees’ fall risk imposes on carers [11].

Like carees, carers also experience fall concern and worry about their carees falling at home. Carers of spouses with Parkinson’s disease expressed loss of confidence and fear when their carees fell [12]. Davey, Wiles, Ashburn and Murphy [12] reported that carers’ concerns go beyond the immediate consequences of falls and encompass the potential impact on carees’ quality of life and survival. Similarly, carers of stroke survivors have fears about their carees falling, especially when they refuse to use prescribed assistive devices [13]. In a grounded theory study, carers of frail carees, the majority suffering from mild cognitive impairment and dementia, expressed fear about them falling [14]. Although carers are generally concerned about carees at risk of falling, the causes of their concern vary across each of the carees’ medical condition.

Falls have significant physical, psychological and social consequences for carers. Some carers reported injuring themselves while trying to prevent carees from falling, or when helping them to get up from the falls [12]. Despite this, carers were reluctant to seek help about their carees’ frequent falling, which places both at further risk of injury [12]. Other carers experienced a significant increase in caregiving burden after their carees had fallen [15, 16]. They described having to change normal routines such as work or social engagements to avoid leaving carees alone at home. This placed the carers at risk of psychological distress and social isolation [16]. The issue of social withdrawal was highlighted among carers of frail carees due to constant worry, vigilance and reluctance to leave their carees alone at home [14]. A cross-sectional study of 96 carers in Australia showed that generally, carers experienced moderate caregiving burden, low self-rated health and poorer quality of life [17]. However, those who looked after carees with high fall risk had significantly greater caregiving burden and depression [17].

Carers may have to change the level of care provided for carees who fall, as they require more assistance, supervision, or on-going care for their fall injuries [16]. The higher level of care needed as the result of falls and fall concern, further increases caregiving burden [13, 16]. When this burden exceeds the carer’s ability to provide adequate care, carees often have no alternative but to be admitted to institutional care. This pathway was explored by Abendroth, Lutz and Young [18] who interviewed twenty primary carers of family members with Parkinson’s disease. For carees who sustained severe injury from falls, this was the main reason for their placement in long-term care.

Most research in this area focuses on carers looking after people with Parkinson’s disease, dementia or stroke, who are at high risk of falling due to functional or cognitive impairment. However, falls do not only affect high risk carees, so it is important to understand more about carers’ concerns, especially those caring for people who are functionally independent but require some form of assistance due to age-related functional or cognitive decline.

There is little quantitative research around this concern. One possible reason is the absence of a reliable instrument to measure carers’ concerns about falls. Studies have quantified the impact of carees’ falling on carers by measuring caregiver burden [15–17], and others have measured the concern for carees falling using a single-item question with binary responses [19], or 10-point Likert scale on how afraid they are of their carees falling [20]. One mixed method study used open-ended questions to assess their perception for fear of falling as there were no validated questionnaires for measuring carers’ fear of falling [11]. Based on the studies reviewed, there is a need for a comprehensive measure of carers’ concern about the fear of carees falling, which includes psychosocial, mental health, quality of life and lifestyle restrictions.

## Study aims and objectives

### Aim

The aim of this study is to explore carers’ fall concern and use this information to develop and test an instrument (Carers’ Fall Concern Instrument [CFC]). As there is no definition for the term carers’ fall concern, we define it as carers’ fear of their carees falling.

### Objectives

The study objectives are to:

1. Identify and describe the different dimensions of carers’ fall concern
2. Develop a pilot instrument to measure these concerns
3. Test the pilot instrument and validate its psychometric properties

## Methods

### Design

This is a mixed method study using an exploratory sequential design to develop the CFC instrument [21]. The study will be conducted over three Phases, beginning with a qualitative approach to explore carers’ fall concern. Building on the qualitative findings, a questionnaire will be constructed and validated. The study has been approved by the Hunter New England Health Human Research Ethics Committee.

### Phase one

A descriptive qualitative study design will be used to explore the phenomena of carers’ concern about carees at risk of falling. It is the method of choice for exploratory research because little is known about carers’ fall concern [22]. This approach provides a comprehensive summary of daily activities using everyday terms and allows researchers to maintain the original data meanings with little interpretation, thus increasing the likelihood of usability and acceptability [23]. After analysis, statements and/or quotes related to carers’ fall concern will be developed into items for the instrument [21]. The multi-item questionnaire will identify carers’ fall concern. Each item will be ranked on a Likert scale of seven categories from strongly disagree, to strongly agree, and a neutral score at middle category [24].

### Phase two

The raw instrument will be reviewed by an expert panel including geriatricians, nurses, allied health professionals and consumers for its face and content validity [25]. Problematic items will be identified for revision and the proposed scoring algorithm of using a seven-point Likert scale will be evaluated. The improved version will then be pilot tested among carers [24]. To ensure comprehensibility and relevance, the target population will involve carers looking after older persons at home with assumption that carers will experience different levels of fall concern. Instrument feasibility and acceptability will be assessed by carers being able to comprehend the questions and willingness to complete the survey [24].

### Phase three

The CFC instrument will be field tested on a larger sample of carers. Exploratory factor analysis will be used to assess factor structure of the instrument [26]. The method consists of defining, extracting, and rotating factors for interpretability, and optimising the dimensionality [24]. Internal consistency (reliability) will be assessed using Cronbach’s alpha to reduce the number of items and improve factor strength. Convergent and discriminant validity will be assessed by comparing the instrument to frequency of carees falling over the past 12 months and injuries sustained to determine if the instrument is measuring what it is intended to measure [24]. Test-retest reliability will be assessed by re-administering the instrument to a subsample of carers two weeks later to ensure that scores received are consistent and stable over time [21]. The evaluation period is chosen to reduce content recall from baseline assessment and changes to events, such as carees’ falling [25].

### Study setting

Participants will be recruited from three study sites: 1) the Hunter Medical Research Institute (HMRI); 2) Carers NSW research register; and 3) the John Hunter Hospital. The HMRI research register is a central database of volunteers living in the Hunter New England Region, Australia, who are keen to participate in medical research as clinical controls [27]. Carers NSW is the peak non-government organisation for carers in New South Wales, Australia and focuses on improving the lives of carers through systemic advocacy and direct carer support [28]. Both databases provide a large cross-section to the general population of carers living in Australia. To enhance discriminatory ability of the CFC instrument, carers of patients from the Rheumatology Outpatient Clinic at John Hunter Hospital will also be recruited. Being the only trauma centre outside Sydney in New South Wales, John Hunter Hospital is a principal referral centre for the Hunter New England Region [29]. As part of osteoporosis re-fracture prevention, patients who are aged above 50 years and admitted to the emergency department for a fracture due to minimal trauma are referred to the Rheumatology Clinic for follow-up. Since most of these fractures are due to falls, the data set of participants from John Hunter Hospital will provide a unique group of carers looking after carees who have had a fall and sustained a fracture for comparison with the general population.

### Sample size

During Phase One, an estimated twenty participants will be recruited using purposive sampling, which involves the deliberate selection of participants, to provide a complete understanding of carers’ concerns [21]. Recruitment will cease upon data saturation. The literature suggests the instrument to be assessed by five to ten experts for its content validity [30]. Another fifty carers will be recruited via convenience sampling for the pilot testing of the CFC instrument [24]. At this stage, it is anticipated that the CFC instrument will contain 20 to 30 items developed from the key concerns shared in the qualitative interviews. The guidelines suggest four to ten participants are needed per item, with a minimum of 100 participants required for exploratory factor analysis [31]. Based on the proportion that 91% of carers were fearful of their carees falling again [19], a sub-sample of 126 carers of carees with an injurious fall is needed for estimating the expected proportion with 5% absolute precision and 95% confidence. It is therefore estimated that 250 participants will need to be recruited during Phase Three, considering that there are carers looking after carees who had not fallen. This sample size is also adequate for eliminating subject variance and identifying adequacy of items in factor analysis [32]. Among the 250 participants, a random sample of fifty participants will be recruited for test-retest of the CFC instrument.

### Inclusion and exclusion criteria

Participants will be eligible if they are: 1) aged above 18 years old; 2) the primary carer for family member or friend; and 3) providing support for at least one personal or instrumental activity of daily living (ADLs). Examples of personal ADLs include mobility, self-care and communication while instrumental ADLs include light housekeeping, transportation and meal preparation. The primary carer is defined in this study as the person who is most involved in caring for a person aged above 60 years old and living at home. The primary carer does not have to live with the caree. Exclusion criteria will include those who were: 1) paid carers or health care providers; 2) being unable to speak English; or, 3) provide informed consent.

### Recruitment process

The coordinators from HMRI and Carers NSW will send out study invitations to their research registry members. At HMRI, those who wish to participate will reply to the HMRI coordinator via a Study Response Form and their contact information will then be forwarded to the researchers for contacting purposes. At Carers NSW, interested participants will need to contact the researchers directly. The study information will be published on HMRI and Carers NSW’s social media such as Facebook page, website and email newsletter.

At the Rheumatology Clinic, the rheumatology nurses will distribute recruitment flyers to the patients for their carers to contact the researchers if they are interested in participating. The nurses will also gather information about carers from the patients and record contact details of patients who are willing to convey the study recruitment to their carers. The researcher will contact all participants to explain the study details, confirm their interest in participating and answer any questions about the research project.

Written Informed Consent will be obtained from Phase One participants. Participation in the survey implies consent for Phase Two and Three. All participants will receive the study information statement, consent form and reply-paid envelope (if applicable) by post or email.

### Data collection methods

During Phase One, carers will be interviewed either face-to-face, or by telephone, depending on their preference, using a semi-structured interview guide. Telephone interview is chosen to allow flexibility for those who are keen to participate in the study, but unable to leave their carees alone at home. The topics to be discussed in the interview will include carers’ concern about carees at risk of falling, factors facilitating care, problems faced during care, personal risks and support received to prevent and manage falls. Follow-up questions and prompts will be used to gain more insight about carers’ fall concern. Demographic data, including age, gender, marital status, employment, care arrangement, history of falls and injury will also be recorded. Each interview session will take approximately one hour and will be audio-recorded. Reflective field notes will also be taken after the interview.

During Phase Two, experts in the area will be asked for their opinion about the accuracy and content relevance of the raw CFC instrument using open-ended questions. They will also rate each item on a four-point Likert scale with one being not relevant and four being very relevant to the construct. Their opinions will be incorporated into an instrument item revision. The revised instrument will then be piloted with 50 carers on-line using Research Electronic Data Capture (REDCap). REDCap is a secure, web-based application designed for building and managing online surveys [33]. It provides an intuitive interface for validated data entry, audit trails for tracking data manipulation and export procedures, automated export procedures for data downloads, and procedures for importing data from external sources [33].

An on-line survey allows carers to complete at their preferred time and provides access to a broad target audience from across the state of NSW. The survey will take approximately 30 min to complete. Participants will complete the CFC instrument and then asked their opinion on the item relevance, demographic questions and falls history of their carees. Findings from the pilot will be used to further refine the instrument. The hyperlink for the on-line survey will be sent to participants via email. For those without email access, the researcher will administer the survey by telephone. A reminder email will be sent to participants after one month to increase response rate.

During Phase Three, the third version of the CFC instrument will be administered to 250 participants. As with Phase Two, a hyperlink to the on-line survey on REDCap will be sent to the participants. The participants will complete the CFC instrument, demographic questions and falls history of their carees. The researcher will administer the survey by telephone for those without email. After two weeks, participants will complete the CFC instrument and report on recent falls history of their carees. A reminder email will be sent if the participants did not complete the online survey after one month.

### Data analysis

During Phase One, analysis of qualitative data will begin simultaneously with data collection to allow researchers to modify data treatment and accommodate new insights [23]. The interviews will be audio-recorded, transcribed verbatim and reviewed for transcription accuracy. An inductive content analysis approach will be applied as little is known about the research topic. The process consists of open coding, forming categories and abstraction [34]. The researcher will write notes and headings in the transcript while reading. Headings will be recorded in coding sheets and grouped to form categories. Repeated patterns in words, phrases, actions or events will identified. During abstraction, these categories are then compared and further categorised to form broader and higher level categories which will be developed into items to describe the hypothetical constructs of carers’ fall concern [34]. Member checking will be conducted to ensure credibility of findings [21, 35].

Feedback regarding the representativeness of CFC instrument will be gathered using open-ended questions from the carers during Phase Two. Method triangulation will be applied to compare the interviews with reflective field notes collected. The researchers’ background and possible influence on the participants’ interaction will be acknowledged to address any potential role conflict. Dependability and confirmability will be ensured by keeping an audit trail which includes the audio recordings, interview transcripts, data analysis documents, and field notes to enhance transparency of research process [35]. To allow transferability, thick description will be used to provide comprehensive illustration of the research context [22].

Quantitative data will be entered into the Statistical Package for Social Science [36] and cleaned to ensure accuracy. The researchers will crosscheck the data with the completed questionnaires to identify any missing values. Frequency tests will be conducted to identify any abnormal values. The data sets will be assumed to be normally distributed.

During Phase Two, the Content Validity Index (CVI) of the proportion of experts rating three and above for each item in the raw CFC instrument will be calculated [25]. Items with CVI score of below 0.80 will be removed. The wordings of the remaining items will also be modified based on the experts’ suggestions.

Descriptive statistics will be used to summarise carers’ demographics, falls history, and data from the CFC instrument. Missing values and distribution item scores will be identified to improve the instrument. An item is considered acceptable if it has less than 3%, but no more than 15% of missing scores [24].

A questionnaire with several missing scores might indicate that participants do not understand the items, do not know the answers, are not willing to provide answers, or items are not applicable [24]. As for item scores distribution, a very high or low mean item score indicates most participants agree or disagree with the item, therefore reducing its power to discriminate. Items with a large percentage of missing scores or low standard deviation will be deleted. Cronbach alpha coefficient will be calculated to assess internal consistency of the raw instrument. Cronbach’s alpha value of between 0.70 and 0.90 is recommended [24, 32]. A Cronbach’s alpha of below 0.70 may indicate too few questions or lack of inter-items homogeneity, while items above 0.90 indicates redundancy of items [37].

During Phase Three, exploratory factor analysis will be conducted with the assumption of normality and homogeneity of variance [26]. Firstly, the suitability of data set for factor analysis will be explored. The use of factor analysis is appropriate if there are substantial numbers of items with a correlation coefficient of above 0.3 [26]. The factorability of data will also be determined by the Bartlett’s test of sphericity and the Kaiser-Meyer-Olkin (KMO) measure of sample adequacy. A *P*-value of < 0.05 for Bartlett’s test and minimum value of 0.6 for KMO show factor analysis to be appropriate [26].

Secondly, factors will be extracted using the principal factor method. The number of factors to be retained will be decided by the criteria of eigenvalue > 1, screen plot test, the proportion of cumulative variance accounted for and the overall interpretability of the factors [24]. Thirdly, factor rotation using either Orthogonal or an Oblique factor solution will be performed to facilitate interpretation of factors for loading closer to 1 or 0 [24]. The selected factors with related items loaded will be labelled [24] and items with a loading below the recommended threshold of 0.4 will be removed [24], as will items with high loadings onto more than one factor. Items will be deleted individually, and factor analysis will be performed after each deletion.

The decision to retain factors of item load with similar eigenvalues will depend on the researchers’ subjective choices regarding content relevance and interpretability of factors. A minimum of three items contributing to each factor is recommended [24]. List-wise deletion will be the primary method of treating missing data, however, if this results in many responses being excluded (greater than 10% of the total sample size), alternative methods will be explored, such as utilising available cases (pairwise-deletion) and multiple imputation. Cronbach alpha coefficient for internal consistency of the instrument is also calculated. Spearman correlation will be used to assess the correlation between carers’ concern and number of falls and severity of injuries sustained. A statistically significant correlation that exceeds 0.5 would suggest the instrument has convergent validity. Discriminant validity will be assessed by using independent T-test to examine group differences in total scores between carers of carees with or without falls and injuries sustained. Test–retest reliability will be assessed by intra-class coefficient between scores obtained during the initial survey and at two weeks’ follow-up.

## Discussion

While there is substantial research about carers’ fall concern for carees suffering from Parkinson’s disease, dementia and stroke, the full picture of carers’ concerns for older people at risk of falling has not been investigated. There is no validated instrument which accurately measures or quantifies this concern. The primary purpose of this study is to develop and validate a measure for carers’ fall concern. This study will explore the different dimensions of carers’ fall concern affecting their physical, psychological and social health, and potentially influence care arrangements for carees.

To develop a self-reported instrument measuring carers’ fall concern, this study will involve the general population of carers looking after older people at home. This will ensure that the items included in the instrument will be important and relevant to carers [38]. Unlike other instruments measuring the psychological impact of falling among older people, the operationalisation of carers’ fall concern is not based upon any theoretical assumptions [39]. Therefore, the instrument will not be limited to activity-related deficits of carees such as in the case of adopting the Self-efficacy Theory. Validity and reliability of the instrument will be ensured by pilot testing with carers and obtaining feedback for modifications from carers and experts.

Several challenges are anticipated in this study. Since the majority of carers are female, there may be a disproportion in gender distribution among the sample population [40]. However, the use of purposive sampling in Phase One and recruitment from research registries will allow access to a diverse population representative of carers. As described in previous studies, most carers worry about leaving their carees alone, and therefore avoid going out of the house [14, 16]. To overcome potential low participation rates among carers, telephone interview will be used for data collection. This will minimise the need for travel and carers may feel more comfortable talking about their experiences and concerns due to the anonymity associated with telephone interview [41].

This study will provide insight into carers’ concerns, promote greater awareness of the psychological impact of caregiving for people at risk of falling, and potentially enable tailored interventions based on carers’ scores on the CFC instrument. As well as measuring carers’ fall concern, the CFC instrument may serve as an alternative measure to predict older persons’ falls risk, therefore overcoming the challenge to assess risk of older people falling, especially those who have cognitive impairment.

A prospective study is proposed to determine if carers’ fall concern would be sensitive to the frequency of carees falling or changes to their medical status and psychosocial health variables. The longitudinal design would also provide clear insight to the causal order between carers’ concern and carees’ subsequent falls. Furthermore, the CFC instrument could also be tested internationally to determine the potential cross-cultural influence of older people falling on carers’ fall concern.

## Abbreviations

ADL: Activity of daily living
Caree: Older person(s)
CFC: Carers’ Fall Concern
CVI: Content Validity Index
HMRI: Hunter Medical Research Institute
KMO: Kaiser-Meyer-Olkin
NSW: New South Wales
REDCap: Research Electronic Data Capture
